# Supplementary material for: Design and validation of a clinical whole genome sequencing-based assay for patient screening in a large healthcare system
Source: Front Mol Biosci. 2025 Oct 13;12:1669085. doi: 10.3389/fmolb.2025.1669085 (PMC12554564; doi:10.3389/fmolb.2025.1669085)
Supplement: Supplementary file 1 [file Supplementaryfile1.pdf]

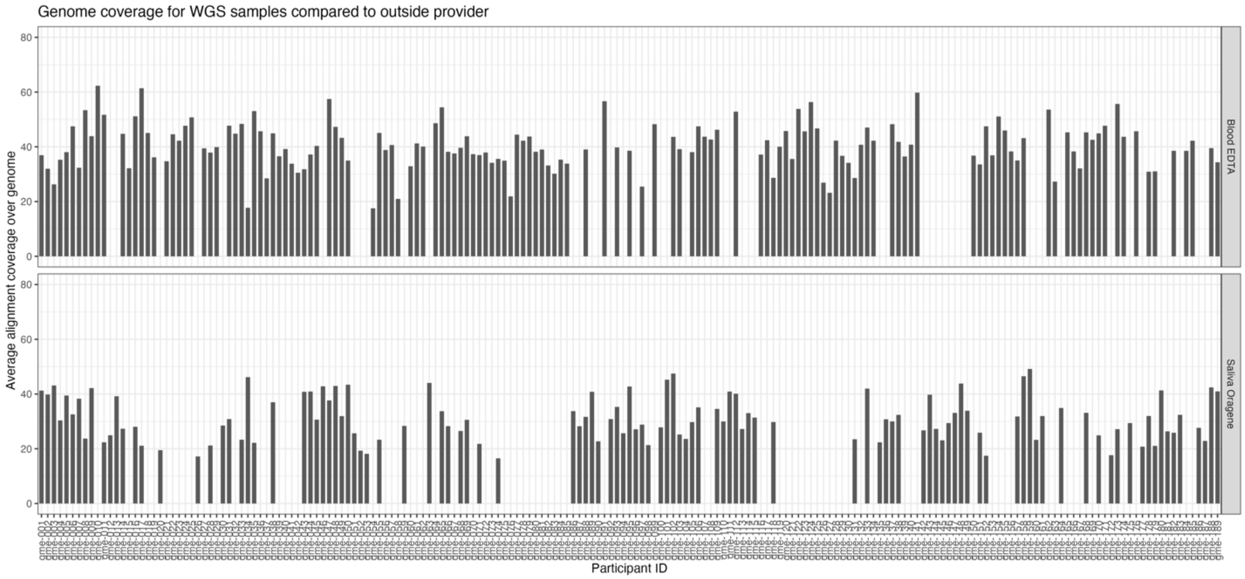

Figure S1 Average alignment coverage over genome for WGS samples in the WGS Validation cohort.

Figure S2. Average alignment coverage over genome for PGx, WES Comparison, and WGS CNV Validation cohorts.

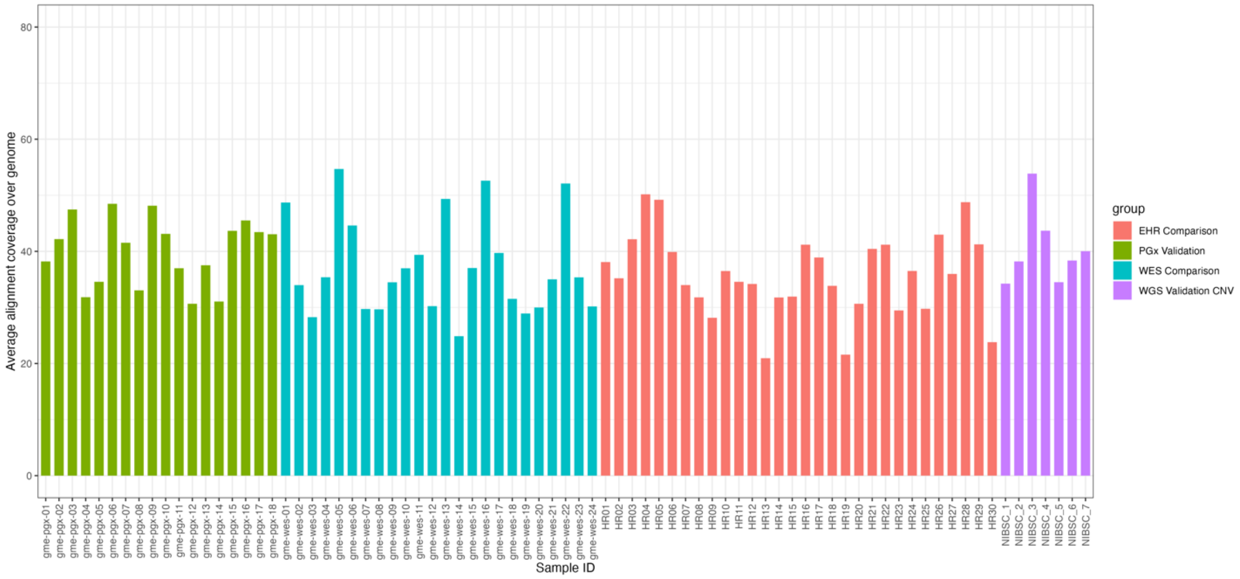

Figure S3. Average alignment coverage over genome (hg19) by method validation group.

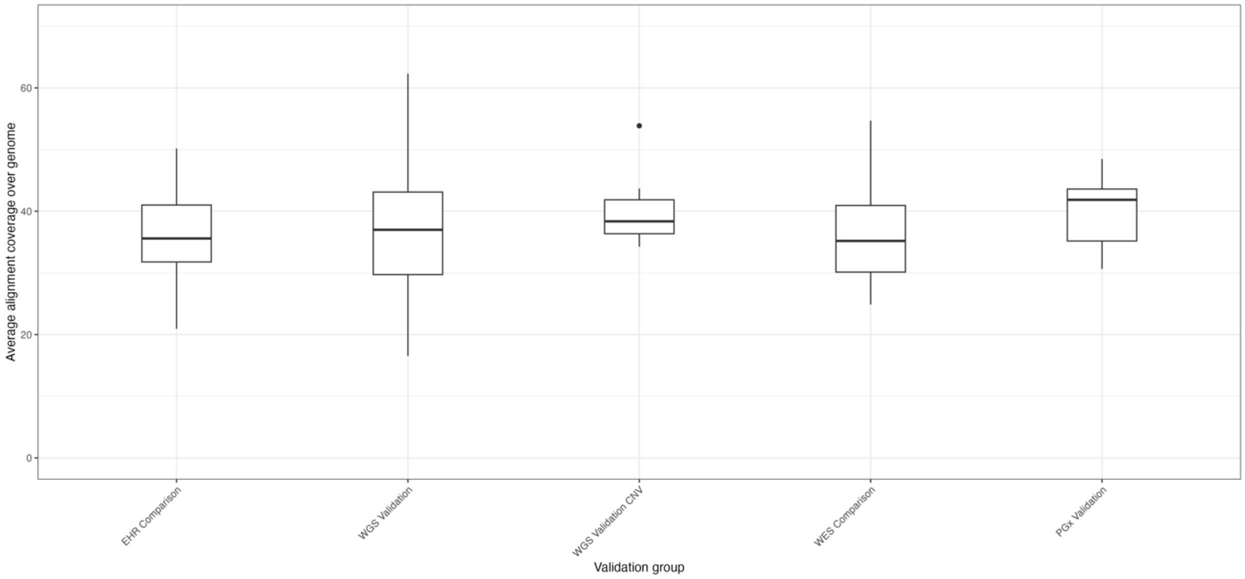

Figure S4. Total number of variants per case that was interpreted by Fabric in the WGS validation cohort.

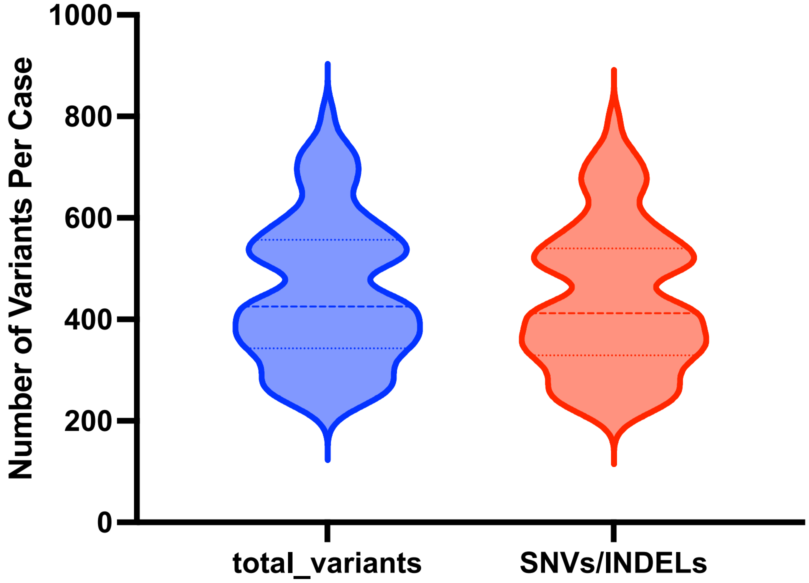

Figure S5. Sensitivity, Specificity calculations for WGS-Fabric method using outside reference method (ORM).

		OUTSIDE REFERENCE METHOD GENE VARIANT DETECTION	
		+	-
WGS-FM GENE VARIANT DETECTION	+	17	0
	-	0	14382

ANALYTIC SPECIFICITY	
True Negative / (False Positive + True Negative)	
True Negative Total	14382
False Positive Total	0
False Positive + True Negative	14382
**Analytical Specificity**	**100.00%**
**95% CI**	**99.97 - 100.00%**

ANALYTIC SENSITIVITY	
True Positive / (True Positive + False Negative)	
True Positive Total	17
False Negative Total	0
True Positive + False Negative	17
**Analytical Sensitivity**	**100.00%**
**95% CI**	**80.49 - 100%**

TECHNICAL ACCURACY	
True Positive + True Negative / (Total Tested)	
True Positive Total	17
True Negative Total	14382
Total Tested	14399
**Technical Accuracy**	**100.00%**
**95% CI**	**99.97 - 100.00%**

NEGATIVE PREDICTIVE VALUE	
True Negative / (True Negative + False Negative)	
True Negative Total	14382
False Negative Total	0
True Negative + False Negative	14382
**Negative Predictive Value**	**100.00%**
**95% CI**	**99.97 - 100.00%**

POSITIVE PREDICTIVE VALUE	
True Positive / (True Positive + False Positive)	
True Positive Total	17
False Positive Total	0
True Positive + False Positive	17
**Positive Predictive Value**	**100.00%**
**95% CI**	**80.49 - 100%**

Figure S6. Sensitivity, Specificity calculations for WGS-Fabric method using WES-ORM.

		REFERENCE ASSAY GENE VARIANT DETECTION	
		+	-
WGS-FM GENE VARIANT DETECTION	+	77	0
	-	1	874

ANALYTIC SPECIFICITY<br>True Negative /<br>(False Positive + True Negative)	
True Negative Total	874
False Positive Total	0
False Positive + True Negative	874
**Analytical Specificity**	**100.00%**
**95% CI**	**99.58 - 100.00%**

ANALYTIC SENSITIVITY<br>True Positive /<br>(True Positive + False Negative)	
True Positive Total	77
False Negative Total	1
True Positive + False Negative	78
**Analytical Sensitivity**	**98.72%**
**95% CI**	**93.06 - 99.97%**

TECHNICAL ACCURACY<br>True Positive + True Negative /<br>(Total Tested)	
True Positive Total	77
True Negative Total	874
Total Tested	952
**Technical Accuracy**	**99.89%**
**95% CI**	**99.42 - 100.00%**

NEGATIVE PREDICTIVE VALUE<br>True Negative /<br>(True Negative + False Negative)	
True Negative Total	874
False Negative Total	1
True Negative + False Negative	875
**Negative Predictive Value**	**99.89%**
**95% CI**	**99.2 - 99.98%**

POSITIVE PREDICTIVE VALUE<br>True Positive /<br>(True Positive + False Positive)	
True Positive Total	77
False Positive Total	0
True Positive + False Positive	77
**Positive Predictive Value**	**100.00%**
**95% CI**	**95.32- 100.00%**

Supplemental Table S1. Outside reference method gene panels (OS-ORM) for genomic conditions surveyed in this study

Gene	Panel	VUS reported
ACTA2	ORM Panel A	no
ACTC1	ORM Panel A	no
APC	ORM Panel A	no
APOB	ORM Panel A	no
ATP7B	ORM Panel A	no
BMPR1A	ORM Panel A	no
BRCA1	ORM Panel A	no
BRCA2	ORM Panel A	no
CACNA1S	ORM Panel A	no
COL3A1	ORM Panel A	no
DSC2	ORM Panel A	no
DSG2	ORM Panel A	no
DSP	ORM Panel A	no
FBN1	ORM Panel A	no
GLA	ORM Panel A	no
KCNH2	ORM Panel A	no
KCNQ1	ORM Panel A	no
LDLR	ORM Panel A	no
LMNA	ORM Panel A	no
MEN1	ORM Panel A	no
MLH1	ORM Panel A	no
MSH2	ORM Panel A	no
MSH6	ORM Panel A	no
MUTYH	ORM Panel A	no
MYBPC3	ORM Panel A	no
MYH11	ORM Panel A	no
MYH7	ORM Panel A	no
MYL2	ORM Panel A	no
MYL3	ORM Panel A	no
NF2	ORM Panel A	no
OTC	ORM Panel A	no
PCSK9	ORM Panel A	no

PKP2	ORM Panel A	no
PMS2	ORM Panel A	no
PRKAG2	ORM Panel A	no
PTEN	ORM Panel A	no
RB1	ORM Panel A	no
RET	ORM Panel A	no
RYR1	ORM Panel A	no
RYR2	ORM Panel A	no
SCN5A	ORM Panel A	no
SDHAF2	ORM Panel A	no
SDHB	ORM Panel A	no
SDHC	ORM Panel A	no
SMAD3	ORM Panel A	no
SMAD4	ORM Panel A	no
STK11	ORM Panel A	no
TGFBR1	ORM Panel A	no
TGFBR2	ORM Panel A	no
TMEM43	ORM Panel A	no
TNNI3	ORM Panel A	no
TNNT2	ORM Panel A	no
TP53	ORM Panel A	no
TPM1	ORM Panel A	no
TSC1	ORM Panel A	no
TSC2	ORM Panel A	no
VHL	ORM Panel A	no
WT1	ORM Panel A	no
APC	ORM Panel B	yes
ATM	ORM Panel B	yes
AXIN2	ORM Panel B	yes
BMPR1A	ORM Panel B	yes
BRCA1	ORM Panel B	yes
BRCA2	ORM Panel B	yes
BRIP1	ORM Panel B	yes
CDH1	ORM Panel B	yes
CDK4	ORM Panel B	yes
CDKN2A	ORM Panel B	yes
CHEK2	ORM Panel B	yes
EPCAM	ORM Panel B	yes
GREM1	ORM Panel B	yes
MEN1	ORM Panel B	yes

MLH1	ORM Panel B	yes
MSH2	ORM Panel B	yes
MSH3	ORM Panel B	yes
MSH6	ORM Panel B	yes
MUTYH	ORM Panel B	yes
NBN	ORM Panel B	yes
NF1	ORM Panel B	yes
NF2	ORM Panel B	yes
NTHL1	ORM Panel B	yes
PALB2	ORM Panel B	yes
PMS2	ORM Panel B	yes
POLD1	ORM Panel B	yes
POLE	ORM Panel B	yes
PTEN	ORM Panel B	yes
RAD51C	ORM Panel B	yes
RAD51D	ORM Panel B	yes
RB1	ORM Panel B	yes
RET	ORM Panel B	yes
SDHAF2	ORM Panel B	yes
SDHB	ORM Panel B	yes
SDHC	ORM Panel B	yes
SDHD	ORM Panel B	yes
SMAD4	ORM Panel B	yes
STK11	ORM Panel B	yes
TP53	ORM Panel B	yes
TSC1	ORM Panel B	yes
TSC2	ORM Panel B	yes
VHL	ORM Panel B	yes
WT1	ORM Panel B	yes
CYP2C19	ORM PGx	no
CYP2C9	ORM PGx	no
CYP4F2	ORM PGx	no
VKORC1	ORM PGx	no

Table S2. Refseq transcripts used by the WGS-FM.

**Gene**	**Transcript**
ACTA2	NM_001613.2
ACTC1	NM_005159.4
APC	NM_000038.5
APOB	NM_000384.2
ATM	NM_000051.3
ATP7B	NM_000053.3
AXIN2	NM_004655.3
BMPR1A	NM_004329.2
BRCA1	NM_007294.3
BRCA2	NM_000059.3
BRIP1	NM_032043.2
CACNA1S	NM_000069.2
CDH1	NM_004360.3
CDK4	NM_000075.3
CDKN2A	NM_000077.4
CHEK2	NM_007194.3
COL3A1	NM_000090.3
DSC2	NM_024422.3
DSG2	NM_001943.3
DSP	NM_004415.2
EPCAM	NM_002354.2
FBN1	NM_000138.4
GLA	NM_000169.2
GREM1	NM_013372.6
KCNH2	NM_000238.3
KCNQ1	NM_000218.2
LDLR	NM_000527.4
LMNA	NM_170707.3
MEN1	NM_130799.2
MLH1	NM_000249.3
MSH2	NM_000251.2
MSH3	NM_002439.4
MSH6	NM_000179.2
MUTYH	NM_001128425.1
MYBPC3	NM_000256.3
MYH11	NM_002474.2
MYH7	NM_000257.2

MYL2	NM_000432.3
MYL3	NM_000258.2
NBN	NM_002485.4
NF1	NM_000267.3
NF2	NM_000268.3
NTHL1	NM_002528.5
OTC	NM_000531.5
PALB2	NM_024675.3
PCSK9	NM_174936.3
PKP2	NM_004572.3
PMS2	NM_000535.5
POLD1	NM_002691.3
POLE	NM_006231.2
PRKAG2	NM_016203.3
PTEN	NM_000314.4
RAD51C	NM_058216.2
RAD51D	NM_002878.3
RB1	NM_000321.2
RET	NM_020975.4
RYR1	NM_000540.2
RYR2	NM_001035.2
SCN5A	NM_198056.2
SDHAF2	NM_017841.2
SDHB	NM_003000.2
SDHC	NM_003001.3
SDHD	NM_003002.3
SMAD3	NM_005902.3
SMAD4	NM_005359.5
STK11	NM_000455.4
TGFBR1	NM_004612.2
TGFBR2	NM_003242.5
TMEM43	NM_024334.2
TNNI3	NM_000363.4
TNNT2	NM_001001430.2
TP53	NM_000546.5
TPM1	NM_001018005.1
TSC1	NM_000368.4
TSC2	NM_000548.3
VHL	NM_000551.3
WT1	NM_024426.4

Table S3. Genotype to phenotype mapping for PGx based on PharmgKB, CPIC, and PharmVar annotations.

**Gene**	**Diplotypes/Genotypes**	**Phenotypes**
CYP2C9	Homozygote or compound heterozygote diplotype including *2, *3, *5, *6, *8, *11	Poor metabolizer (PM)
	Heterozygote for *2, *3, *5, *6, *8, *11	Intermediate metabolizer (IM)
	None of the tested alleles (*2, *3, *5, *6, *8, *11) detected. Reported as *1/*1	Normal metabolizer (NM)
CYP2C19	Homozygote or compound heterozygote diplotype including two no function alleles (*2, *3, *4A, *4B, *5, *6, *7, *8, *35)	Poor metabolizer (PM)
	Compound heterozygote diplotype including one decreased function allele (*9 or *10) and one no function allele (*2, *3, *4A, *4B, *5, *6, *7, *8, and *35)	Likely poor metabolizer (likely PM)
	Compound heterozygote diplotype including *17 or *1 (i.e. no *17 detected), and one no function allele (*2, *3, *4A, *4B, *5, *6, *7, *8, and *35)	Intermediate metabolizer (IM)
	Compound heterozygote diplotype including *17 or *1 (i.e. no *17 or other tested alleles detected), and one decreased function allele (*9 or *10)<br>OR<br>Homozygote or compound heterozygote diplotype including two decreased function alleles (*9 and *10)	Likely intermediate metabolizer (Likely IM)
	None of the tested alleles (*2, *3, *4A, *4B, *5, *6, *7, *8, *9, *10, *17, *35) detected. Reported as *1/*1	Normal metabolizer (NM)
	Heterozygote for only *17 (no other tested alleles detected). Reported as *1/*17	Rapid metabolizer (RM)
	Homozygote for *17	Ultra rapid metabolizer (UM)
CYP4F2	Heterozygote or homozygote for *3	Warfarin resistant
	No *3 detected. Reported as *1/*1	Normal warfarin sensitivity
VKORC1 (c.-1639G>A, rs9923231)	GA or AA	Warfarin sensitive
	GG	Normal warfarin sensitivity
	GA, AA	Warfarin sensitive

rs12777823 (CYP2C gene cluster)	GG	Normal warfarin sensitivity
